# Adult T-Cell Lymphoma/Leukemia Presenting as Isolated Central Nervous System T-Cell Lymphoma

**DOI:** 10.1155/2014/917369

**Published:** 2014-12-23

**Authors:** Wei-Li Ma, Chi-Cheng Li, Shan-Chi Yu, Hwei-Fang Tien

**Affiliations:** ^1^Department of Oncology, National Taiwan University Hospital, Yun-Lin Branch, Yun-Lin 64041, Taiwan; ^2^Division of Hematology, Department of Internal Medicine, National Taiwan University Hospital, Taipei 10002, Taiwan; ^3^Tai-Cheng Stem Cell Therapy Center, National Taiwan University Hospital, 7 Chung-Shan South Road, Taipei 10002, Taiwan; ^4^Department of Pathology, National Taiwan University Hospital, Taipei 10002, Taiwan

## Abstract

Adult T-cell leukemia/lymphoma (ATLL) is a T-cell neoplasm, associated with infection by the retrovirus human T-lymphotropic virus type 1 (HTLV-1). Central nervous system (CNS) involved by ATLL is often occurred in advanced disease, such as acute and lymphomatous variants. On the other hand, isolated CNS lymphoma is rare. We repot a 50-year-old woman who presented with multiple infiltrative brain lesions on the magnetic resonance (MR) imaging. Results of initial biopsy of brain tumor indicated CNS vasculitis. The patient received one course of high-dose methotrexate and MR imaging of brain revealed remission of infiltrative lesions. Two years later, new brain lesions were detected. Histopathologic examination of specimens via craniotomy revealed T-cell lymphoma. The patient responded poorly to subsequent chemotherapy, and salvage whole-brain irradiation was performed. Six months later, the patient had hepatosplenomegaly, hypercalcemia, and multiple lymphocytes with a cloverleaf appearance in circulation. Results of flow cytometry analysis of peripheral blood indicated ATLL and antibodies to human T-lymphotropic virus type 1 (HTLV-1) were detected. Clinicians should screen HTLV-1 infection when patients are diagnosed with peripheral T-cell lymphoma. Combined antiviral therapy and intensive chemotherapy may improve the outcomes of ATLL.

## 1. Introduction

ATLL is an aggressive malignancy of activated mature T lymphocytes caused by the retrovirus HTLV-1. The disease is resistant to multiple chemotherapy agents and is characterized by severe immunosuppression, resulting in poor survival [1]. Acute, lymphomatous, chronic, and smoldering variants of ATLL have been identified. CNS involvement is more common in the lymphomatous and acute forms [2, 3]. Herein, we report a rare case of ATLL that was originally diagnosed as isolated CNS T-cell lymphoma. Despite multiple chemotherapy sessions, the brain lesions progressed and the acute variant of ATLL was diagnosed 3 years after the patient first presented with symptoms. Patients in whom T-cell lymphoma is diagnosed should be screened for the presence of HTLV-1 antibodies, even without abnormal lymphoid cells in circulation.

## 2. Case Presentation

**Figure 1:**
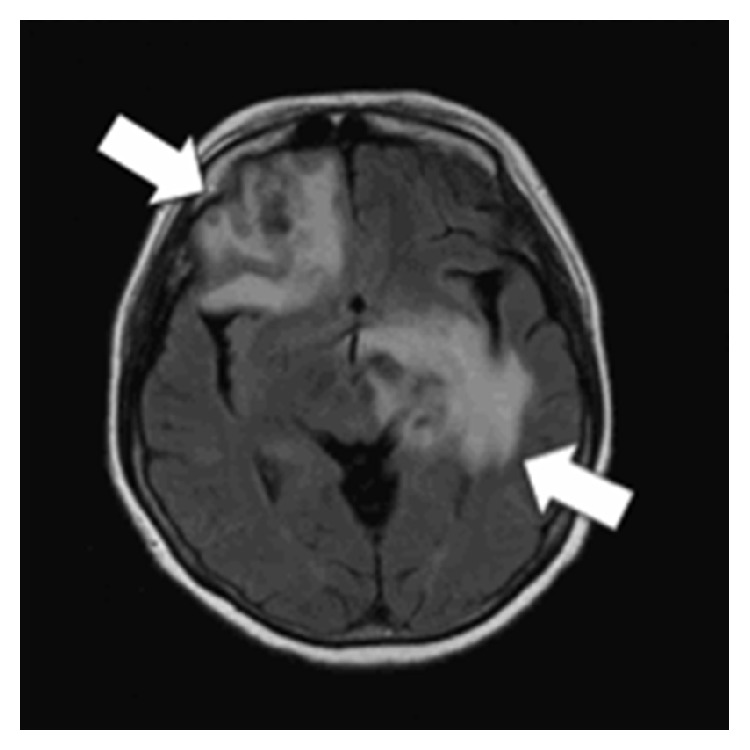
Fluid attenuation inversion recovery- (FLAIR-) weighted MR imaging of brain with contrast showed multiple infiltrative lesions (arrows) in the left basal ganglion, left thalamus, and right frontal periventricular white matter.

A previously healthy 50-year-old woman presented with a multiple-month history of intractable headache and dizziness. The MR imaging of brain revealed infiltrative lesions in the left basal ganglion, left thalamus, and right frontal periventricular white matter, with minimal internal enhancement (Figure 1). A preliminary diagnosis of focal gliosis was made based on the result of the stereotactic biopsy procedure. One month later, however, the brain lesions progressed and the patient underwent open biopsy. Microscopic examination revealed necrosis and gliosis of brain tissue with perivascular inflammatory cell infiltration. The inflammatory cells stained positive for CD3 and CD20. CNS vasculitis was suspected but lymphoma could not be completely excluded. The computed tomography (CT) of the chest, abdomen, and pelvic bone showed no systemic lymphadenopathy. The patient did not have abnormal lymphoid cells in peripheral blood, bone marrow, and cerebrospinal fluid, and her serum calcium was within reference range. One cycle of high-dose methotrexate (6 g/m^2^ on day 1) with leucovorin rescue was administered for the vasculitis or undiagnosed lymphoma. Her neurologic signs improved, and the follow-up MR imaging of brain showed decreased size and mass effect of the brain lesions. However, the patient had recurrent bacteremia, osteoarthritis, and necrotizing fasciitis after the chemotherapy.

**Figure 2:**
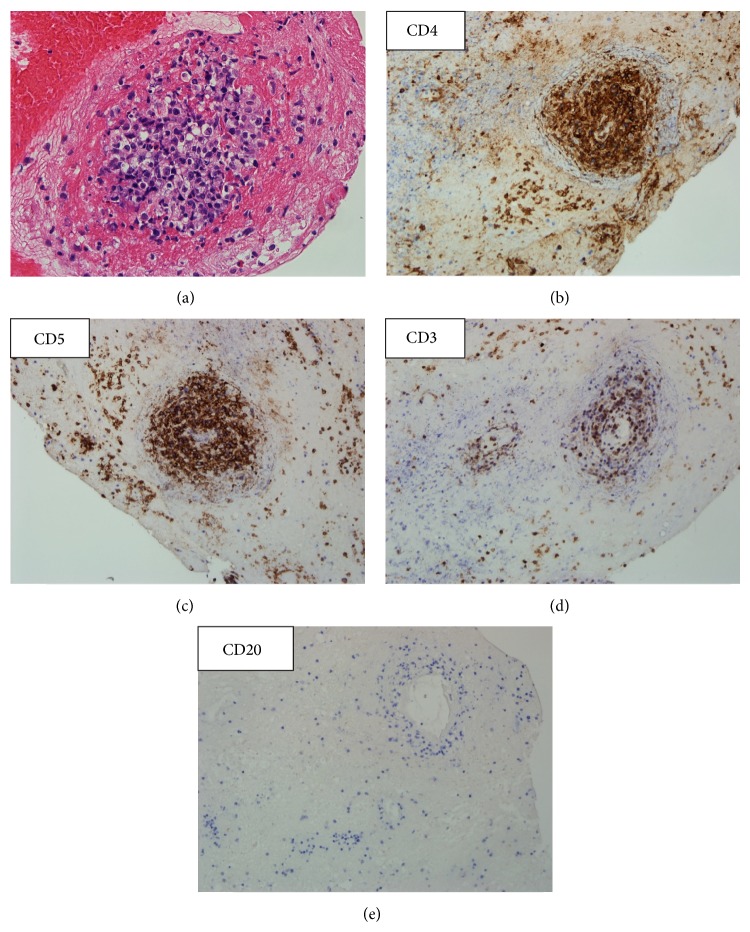
Microscopic findings of CNS T-cell lymphoma. Hematoxylin and eosin stain, 1000x. The lesions showed tissue necrosis and perivascular infiltration of atypical, medium-sized lymphoid cells (a). The lymphoid cells had strong membrane staining for CD4 (b), CD5 (c), focal positive for CD3 (d), and negative for CD20 (e).

**Figure 3:**
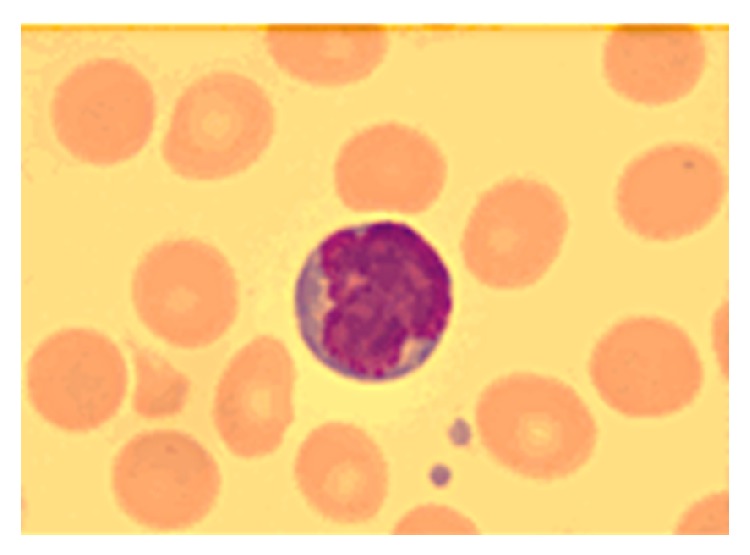
Liu stain of peripheral blood smear, 1000x. Multiple lymphocytes with a cloverleaf appearance, the so called “flower-like” cells, were noted.

Two years later, the patient presented with right-sided hemiparesis and her brain lesions had progressed, with enlarged size and prominent perifocal edema. She received a biopsy via craniotomy. Microscopically, the atypical lymphoid cells showed perivascular infiltration with positive staining for CD45, CD5, CD4, CD8 and focally for CD3 (Figure 2). Primary CNS T-cell lymphoma was diagnosed, and there was no extracranial involvement. She received four cycles of high-dose methotrexate (8 g/m^2^ on day 1) with leucovorin rescue but her brain disease progressed. Salvage chemotherapy comprising BAS (carmustine 65 mg/m^2^ on day 1 and day 2; cytarabine 2000 mg/m^2^ on day 1; methylprednisolone 200 mg on days 1–5) was administered but her neurologic symptoms still deteriorated. Finally, she received whole-brain radiation therapy (WBRT) (30 Gray/15 fractions) to control her disease. Six months later, the patient was admitted due to fever and hepatosplenomegaly. In addition, she also had abnormal lymphocytes with a cloverleaf appearance in peripheral blood (Figure 3), hypercalcemia, and positive serum antibody to HTLV-1. The results of flow cytometry analysis of peripheral blood supported the diagnosis of adult T-cell leukemia, with the expression of CD2, CD3, CD4, CD5, and CD25 but no expression of CD7. A brain tumor specimen was sent to the National Cancer Institute in America for further examination after disease progression. T-cell receptor (TCR) rearrangement was detected, thereby supporting the diagnosis of T-cell lymphoma. In addition, reverse transcription polymerase chain reaction (RT-PCR) yielded HTLV-1 deoxyribonucleic acid (DNA) sequences in brain tissue. Those two findings confirmed the diagnosis of ATLL with isolated CNS involvement. However, the patient died due to severe septic shock before receiving any treatment for ATLL.

## 3. Discussion

HTLV-1 is endemic in populations in southern Japan, Africa, the Caribbean, and South America [4]. It is not endemic in Taiwan, and the prevalence is about 0.48% [5]. The immunophenotype of tumor cells is CD3+, CD7−, CD4+/CD8−, and CD25+. A few cases have been shown to be CD4−, CD8+ or double positive for CD4 and CD8 [6]. This indicates that the malignancy derives from activated helper T cells and explains the immunodeficiency of our patient, who had multiple episodes of infection after the first cycle of chemotherapy despite a lack of evidence for myelosuppression during treatment.

CNS involvement in ATLL occurs in up to 25% of cases. The characteristic findings on image studies are multiple parenchymal lesions, with or without enhancement, in the deep grey matter of both hemispheres and leptomeningeal lesions. Compared with primary CNS lymphoma, ATLL lesions in the CNS tend to spread more extensively but are enhanced less frequently [7]. Microscopically, the malignant lymphocytes infiltrate the meninges and perivascular space. The systemic progression of ATLL was the most common setting of CNS involvement and the major cause of death [2]. However, cases of isolated CNS ATLL without systemic involvement have also been reported with poor prognosis [8–11]. In addition, ATLL has been shown to be related to Epstein-Barr virus-associated CNS lymphoma, JC virus-associated multifocal leukoencephalopathy, human herpesvirus, and toxoplasmatic encephalitis [12–15]. Thus, HTLV-1 should be screened in all patients with T-cell lymphoma or opportunistic infection in brain even when there is no evidence for atypical lymphocytes in circulation.

Primary CNS lymphoma is primarily treated with a high-dose systemic methotrexate-based regimen [16]. Other CNS-penetrant chemotherapies, such as cytarabine and carmustine, in combination with methotrexate have been shown to increase the treatment response [17, 18], although the optimal combination is uncertain. WBRT can be a highly effective treatment [19], and radiotherapy consolidation after chemotherapy can improve survival [20]. However, the WBRT in our patient was palliative, because multiple CNS-penetrant drug therapies were unable to control her progressive brain lymphoma. Combined antiviral Zidovudine (AZT) and interferon-alpha have been shown to have good response and to prolong survival [21]. Thus, if ATLL had been diagnosed earlier in our patient, combined AZT and interferon-alpha may have improved her outcome.

In conclusion, HTLV-1 infection should be evaluated in patients with T-cell lymphoma or opportunistic infections in brain, even in nonendemic areas, such as Taiwan. Although AZT and interferon-alpha improve the survival of patients with ATLL, more effective treatments are necessary, especially for patients who cannot tolerate intensive chemotherapy.

## References

[1] Bazarbachi A., Ghez D., Lepelletier Y., Nasr R., de Thé H., El-Sabban M. E., Hermine O. (2004). New therapeutic approaches for adult T-cell leukaemia. *The Lancet Oncology*.

[2] Teshima T., Akashi K., Shibuya T. (1990). Central nervous system involvement in adult T-cell leukemia/lymphoma. *Cancer*.

[3] Kawasaki C., Ikeda H., Fukumoto T. (1995). Cerebral mass lesions associated with adult T-cell leukemia/lymphoma. *International Journal of Hematology*.

[4] Bazarbachi A., Suarez F., Fields P., Hermine O. (2011). How I treat adult T-cell leukemia/lymphoma. *Blood*.

[5] Wang C.-H., Chen C.-J., Hu C.-Y., You S.-L., Chu C.-T., Chou M.-J., Essex M., Blattner W. A., Liu C.-H., Yang C.-S. (1988). Seroepidemiology of human T-cell lymphotropic virus type I infection in Taiwan. *Cancer Research*.

[6] Ohshima K., Jaffe E. S., Kikuchi M., Swerdlow S. H. (2008). Adult T-cell leukemia/lymphoma. *WHO Classification of Tumours of Haematopoietic and Lymphoid Tissue*.

[7] Kitajima M., Korogi Y., Shigematsu Y., Liang L., Matsuoka M., Yamamoto T., Jhono M., Eto K., Takahashi M. (2002). Central nervous system lesions in adult T-cell leukaemia: MRI and pathology. *Neuroradiology*.

[8] Marshall A. G., Pawson R., Thom M. (1998). HTLV-I associated primary CNS T-cell lymphoma. *Journal of Neurological Sciences*.

[9] Dungerwalla M., Osuji N., Waldman A. D. (2005). Isolated central nervous system involvement in adult T-cell lymphoma/leukaemia. *British Journal of Haematology*.

[10] Komuro T., Okamoto S. (2010). Pure intracerebral mass lesion of adult T-cell leukemia/lymphoma—case report. *Neurologia Medico-Chirurgica*.

[11] Lotan I., Khlebtovsky A., Inbar E., Strenov J., Djaldetti R., Steiner I. (2012). Primary brain T-cell lymphoma in an HTLV-1 serologically positive male. *Journal of the Neurological Sciences*.

[12] Amano M., Marutsuka K., Sugimoto T., Todaka T., Setoyama M. (2011). Epstein-Barr virus-associated primary central nervous system lymphoma in a patient with adult T-cell leukemia/lymphoma. *The Journal of Dermatology*.

[13] Kimura A., Ueyama H., Kimura N., Fujimoto S., Kumamoto T. (2006). Progressive multifocal leukoencephalopathy in an HTLV-I carrier. *Clinical Neurology and Neurosurgery*.

[14] Idutsu K., Abe Y., Otonari J., Tachikawa Y., Ohtsuka R., Choi I., Muta K., Takayanagi R. (2007). Human herpesvirus 6 encephalitis in a patient with adult T-cell leukemia/lymphoma. *Rinsho Ketsueki*.

[15] Re D., Reiser M., Bamborschke S., Schröder R., Lehrke R., Tesch H., Salzberger B., Diehl V., Fätkenheuer G. (1999). Two cases of toxoplasmic encephalitis in patients with acute T-cell leukaemia and lymphoma. *The Journal of Infection*.

[16] Batchelor T., Carson K., O'Neill A. (2003). Treatment of primary CNS lymphoma with methotrexate and deferred radiotherapy: a report of NABTT 96-07. *Journal of Clinical Oncology*.

[17] Ferreri A. J., Reni M., Foppoli M., Martelli M., Pangalis G. A., Frezzato M., Cabras M. G., Fabbri A., Corazzelli G., Ilariucci F., Rossi G., Soffietti R., Stelitano C., Vallisa D., Zaja F., Zoppegno L., Aondio G. M., Avvisati G., Balzarotti M., Brandes A. A., Fajardo J., Gomez H., Guarini A., Pinotti G., Rigacci L., Uhlmann C., Picozzi P., Vezzulli P., Ponzoni M., Zucca E., Caligaris-Cappio F., Cavalli F. (2009). High-dose cytarabine plus high-dose methotrexate versus high-dose methotrexate alone in patients with primary CNS lymphoma: a randomised phase 2 trial. *The Lancet*.

[18] Cheng A.-L., Yeh K.-H., Uen W.-C., Hung R.-L., Liu M.-Y., Wang C.-H. (1998). Systemic chemotherapy alone for patients with non-acquired immunodeficiency syndrome-related central nervous system lymphoma: a pilot study of the BOMES protocol. *Cancer*.

[19] Nelson D. F., Martz K. L., Bonner H., Nelson J. S., Newall J., Kerman H. D., Thomson J. W., Murray K. J. (1992). Non-Hodgkin's lymphoma of the brain: can high dose, large volume radiation therapy improve survival? Report on a prospective trial by the Radiation Therapy Oncology Group (RTOG): RTOG 8315. *International Journal of Radiation Oncology Biology Physics*.

[20] DeAngelis L. M., Seiferheld W., Clifford Schold S., Fisher B., Schultz C. J. (2002). Combination chemotherapy and radiotherapy for primary central nervous system lymphoma: radiation Therapy Oncology Group study 93–10. *Journal of Clinical Oncology*.

[21] Gill P. S., Harrington W., Kaplan M. H., Ribeiro R. C., Bennett J. M., Liebman H. A., Bernstein-Singer M., Espina B. M., Cabral L., Allen S., Kornblau S., Pike M. C., Levine A. M. (1995). Treatment of adult T-cell leukemia-lymphoma with a combination of interferon alfa and zidovudine. *The New England Journal of Medicine*.

